# Supplementary material for: Study on the evolution of the spatial patterns and driving factors of national agricultural cultural heritage in China
Source: PLoS One. 2025 Jan 24;20(1):e0313926. doi: 10.1371/journal.pone.0313926 (PMC11761124; doi:10.1371/journal.pone.0313926)
Supplement: Attachment — Submitted filename: Review comments.docx [file pone.0313926.s002.docx]

In this paper, spatial analysis methods such as the nearest-neighbor index, center-of-gravity-standard deviation ellipse, kernel density, spatial autocorrelation and geographic probes are used to explore the spatial distribution characteristics and driving factors of national agricultural cultural heritage. Generally, the structure of the paper is clear and complete, but the theoretical depth is general, and there is still room for improvement in the depth and breadth of the research. Here are some comments:

1.Abstract. The description of the study context/importance of the study is relatively good, the study methods and data are clearly described. However, if possible，these parts should be more simply and the main findings part should be more accurate and detailedly.

2.The introduction should outline the background of the undertaken research. However, the research gap is not discussed deeply enough. Increase the summary and brief review of relevant research literature, and highly refine the shortcomings of research and the main innovations or contributions of this manuscript.

3.Figures. The format of the figures should carefully refer to the journal requirements. Some words in the picture cannot be read clearly. Tables. The format of the tables should carefully refer to the journal requirements.

4.The layout of the full text (Including references)is very rough, and it is suggested that the author should carefully revise and improve according to the standards of the journal.
